# Visualizing real-time influenza virus infection, transmission and protection in ferrets

**DOI:** 10.1038/ncomms7378

**Published:** 2015-03-06

**Authors:** Erik A. Karlsson, Victoria A. Meliopoulos, Chandra Savage, Brandi Livingston, Andrew Mehle, Stacey Schultz-Cherry

**Affiliations:** 1 Department of Infectious Diseases, St Jude Children’s Research Hospital, Memphis, Tennessee 38105, USA; 2 Animal Resource Center, St Jude Children’s Research Hospital, Memphis, Tennessee 38105, USA; 3 Department of Medical Microbiology and Immunology, University of Wisconsin-Madison, Madison, Wisconsin 53706, USA

## Abstract

Influenza transmission efficiency in ferrets is vital for risk-assessment studies. However, the inability to monitor viral infection and transmission dynamics in real time only provides a glimpse into transmissibility. Here we exploit a replication-competent influenza reporter virus to investigate dynamics of infection/transmission in ferrets. Bioluminescent imaging of ferrets infected with A/California/04/2009 H1N1 virus (CA/09) encoding NanoLuc (NLuc) luciferase provides the first real-time snapshot of influenza infection/transmission. Luminescence in the respiratory tract and in less well-characterized extra-pulmonary sites is observed, and imaging identifies infections in animals that would have otherwise been missed by traditional methods. Finally, the reporter virus significantly increases the speed and sensitivity of virological and serological assays. Thus, bioluminescent imaging of influenza infections rapidly determines intra-host dissemination, inter-host transmission and viral load, revealing infection dynamics and pandemic potential of the virus. These results have important implications for antiviral drug susceptibility, vaccine efficacy, transmissibility and pathogenicity studies.

## Abstract (web summary)

Ferrets are the main animal model used for research on influenza transmission. Here, the authors investigate the dynamics of infection and transmission in ferrets using a replication-competent influenza reporter virus and real-time bioluminescence imaging.

## Graphical abstract

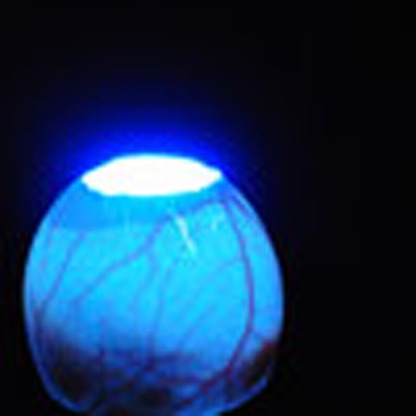

Seasonal outbreaks of influenza viruses are associated with millions of cases of severe illness and 250,000–500,000 deaths each year^1^. In addition, the last decade has seen multiple human infections with zoonotic influenza A viruses from swine and avian reservoirs, sometimes with serious clinical consequences^2^,^3^. Infections of humans with seasonal influenza viruses typically involve upper respiratory tract (URT) infections; however, pandemic and zoonotic viruses tend to be more pathogenic, possibly due to increased tropism for the lower respiratory tract (LRT)^4^. Recent studies have highlighted that viral dynamics and spread can greatly impact respiratory disease severity, transmissibility and protection from re-infection^5^. Indeed, the ability to efficiently replicate in the URT is considered to be necessary to confer transmissibility^6^. Unfortunately, conventional animal studies are unable to monitor real-time viral dynamics or patterns of infection and are constrained to monitoring survival or clinical signs of infection, requiring euthanasia to collect tissues for the assessment of viral infection or therapeutic efficacy^7^,^8^,^9^.

Bioluminescent reporter systems afford the ability to visualize intra- and inter-host dynamics in real-time. Although multiple influenza reporter viruses have been generated^10^,^11^,^12^,^13^,^14^, the majority are significantly attenuated and/or genetically unstable compared with parental strains. We recently described an exquisitely sensitive influenza reporter virus that stably encodes the engineered luciferase variant NanoLuc (NLuc)^15^ in the polymerase (PA) gene segment. This virus replicates at near-wild-type levels and recapitulates known restrictions due to host range and antiviral treatment, permitting imaging of sublethal infections and clearance in mice^16^. The ferret is the preferred, ‘gold standard’ model for the study of influenza virus pathogenicity and transmission^17^,^18^, yet it is not clear if bioluminescent imaging is suitable for transmission studies in larger animals. Here we report the application of this technology to a primary viral isolate, the pandemic 2009 H1N1 (A/California/04/2009; CA/09) strain, to track real-time replication and transmission as well as visualizing differential replication patterns that accurately reflect viral replication despite the size of the ferret model. This approach has the potential to assess the tissue distribution, transmissibility and pandemic potential of emerging influenza viruses in larger animal models.

## Results

### Native replication of CA/09-PA NLuc virus *in vitro*

**Figure 1:**
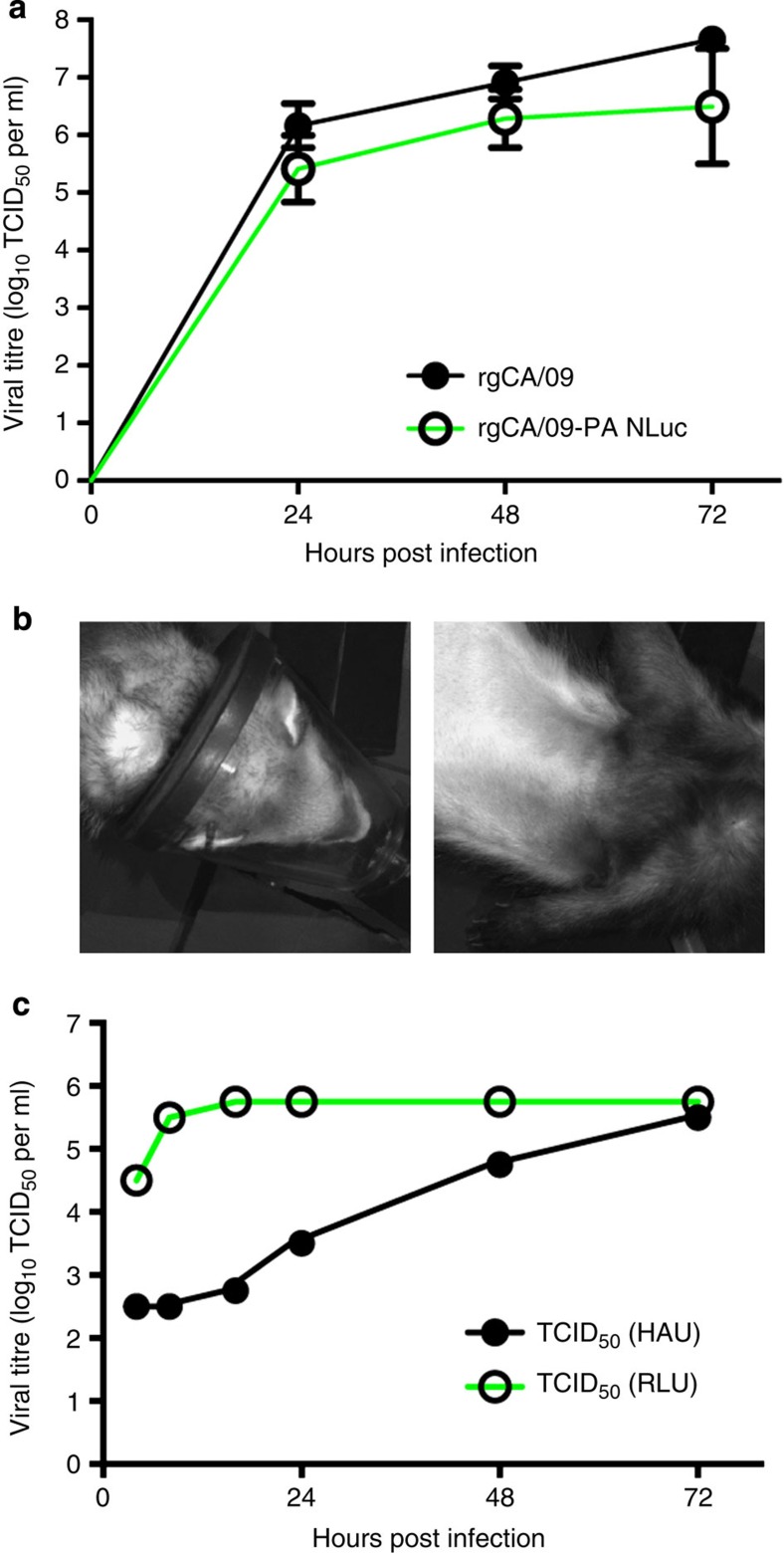
Validation of CA/09-PA NLuc virus against parental rgCA/09 virus. (**a**) Replication kinetics of the CA/09-PA NLuc virus were compared with parental rgCA/09 virus by TCID_50_ analysis on MDCK cells. Samples were run in triplicate, Error bars=s.d. (**b**) Eight-week-old male ferrets were lightly anaesthetised and intranasally inoculated with 10^5^ TCID_50_ rgCA/09 virus. Animals were administered 200 μl substrate in 1 ml PBS via the cephalic vein and images taken over a 4-min exposure. No luminescence is seen since the virus does not contain the swapped PA NLuc gene. (**c**) Comparison of time to maximal titre in MDCK cells between luminescent and traditional hemagglutination (HAU) end points in ferret nasal wash from triplicate samples.

To assess the utility of the NLuc reporter virus in ferrets, a replication-competent NanoLuc A/California/04/2009 (CA/09-PA NLuc) virus was generated by inserting NanoLuc into the polymerase gene as described^16^. We successfully rescued the CA/09-PA NLuc virus by transfecting 293T cells using an eight-plasmid reverse genetics system and propagating the resultant virus in embryonated chicken eggs^19^. Virus propagation through three passages in eggs did not result in attenuation of viral luminescence, suggesting stable maintenance of the reporter construct, and the replication kinetics of the reporter virus are indistinguishable from the parental strain *in vitro* (Fig. 1a).

### Imaging real-time dynamics and viral replication patterns

**Figure 2:**
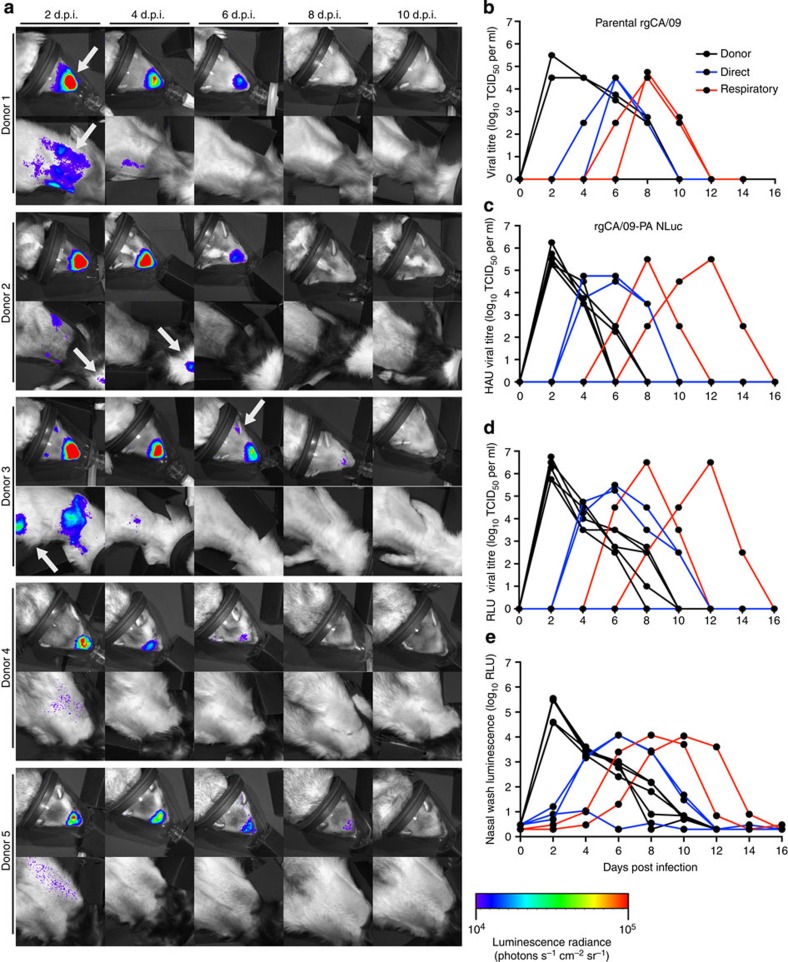
CA/09-PA NLuc reporter virus has similar dynamics of transmission as rgCA/09 parental virus. (**a**) Ferrets (*n*=5) were directly inoculated with CA/09-PA NLuc reporter virus. Upper respiratory and lung bioluminescence was imaged every 48 h.p.i. through 14 d.p.i. Imaging ended when viral clearance was obtained as monitored by both luminescence and viral titre determination. Arrows indicate areas of interest where luminescence was detected. (**b**) Nasal washes from donor ferrets directly inoculated with parental rgCA/09 (black lines, *n*=5) virus, direct contacts (blue lines, *n*=3) and respiratory contact ferrets (red lines, *n*=2) were titrated by TCID_50_ assay. (**c**–**e**) Nasal washes from ferrets directly inoculated with rgCA/09-PA NLuc virus (black lines) or direct (blue lines) and respiratory contact (red lines) animals were titrated by TCID_50_ assay using HAU as an end point (**c**), luminescent TCID_50_ assay (**d**) or the nasal wash was directly assessed for luminescence (**e**). Lines represent individual animals.

To test whether CA/09-PA NLuc measures viral infection dynamics in real time and is suitable for transmission studies, donor ferrets (*n*=5) were inoculated with parental CA/09 virus (Fig. 1b) or CA/09-PA NLuc virus and serially imaged throughout infection (Fig. 2a). Nasal washes were collected from 2 to 14 days post infection (d.p.i.) and viral titres, as measured by traditional TCID_50_ analysis, demonstrated that all the donor parental- and NLuc-inoculated animals became infected with titres peaking at ~10^6^ TCID_50_ (Fig. 2b,c). Titres were comparable between CA/09 and CA/09-PA NLuc viruses (Fig. 2b,c, Table 1), demonstrating that the reporter virus replicates similarly to the parental, and titres mirror previously reported studies using pdmH1N1 viruses in ferrets^20^,^21^,^22^,^23^. A TCID_50_ assay with a bioluminescent endpoint was also developed (Fig. 2d). The bioluminescent TCID_50_ (RLU) assay yielded titres nearly identical to the classical TCID_50_ (HAU) assay, but dramatically accelerated determination of viral titre requiring only 18 h compared with TCID_50_ (HAU) measurements that require 72 h (Fig. 1c, Table 1). Finally, we directly measured NLuc activity in the nasal washes (Fig. 2e). Luminescence in nasal wash was significantly correlated to viral titres determined by both TCID_50_ assays and thus permits instantaneous measurement of changes in viral load (Fig. 2e). Significant correlation between nasal wash luminescence and viral titre was confirmed using Pearson’s correlation coefficient (Table 1).

**Figure 3:**
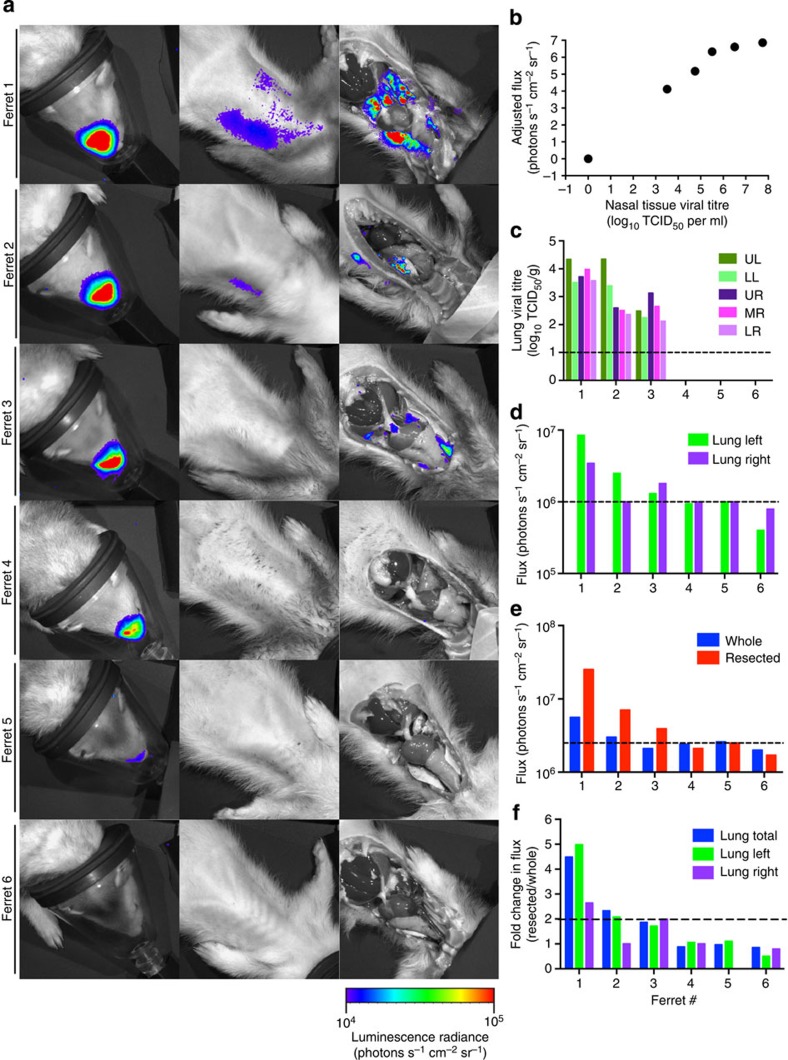
Correlation of viral titre and bioluminescence in CA/09-PA NLuc infected ferrets. (**a**) Ferrets (*n*=6) infected with CA/09-PA NLuc were imaged whole and resected. (**b**) Viral titres from nasal tissue were significantly correlated with nasal flux. (**c**) Viral load was measured in each lung lobe (UL, upper left; LL, lower left; UR, upper right; MR; middle right; LR, lower right) above the limit of detection (dashed line). Increased flux was associated with higher viral titres above background (dashed line) when data were considered for the right or left lobes (**d**) and whole lung (**e**). (**f**) Comparison of bioluminescence between whole and resected animals showed that overlying tissue can reduce flux from the lungs up to 4.5-fold. Bars represent individual animals.

Imaging also revealed heterogeneous tissue distribution in influenza virus-infected ferrets (Fig. 2a). Bioluminescence was detected in the URT and LRT of all directly inoculated (donor) animals, indicating infection in the nasal turbinates and lungs respectively. All animals showed infection in the URT by 2 d.p.i., waning by 4–6 d.p.i. and resolving to below the level of detection by 8 d.p.i. Robust bioluminescence was detected in both the URT and LRT for two of the animals (donors 1 and 3). Multifocal bioluminescence detected in the LRT 2 and 4 d.p.i. is consistent with infection in multiple lobes of the lung and the trachea. Low-level infection was detected in the lungs of donor 2 that was completely resolved by 4 d.p.i. In contrast, there was significantly less lung infection evident in donors 4 and 5. Imaging of resected animals confirmed the ability to accurately localize URT and LRT infections as well as distinguish infections in the left and right lobes of the lung (Fig. 3a).

### Bioluminescent detection correlates with titres

Luminescent flux (radiance defined as photons s^−1^ cm^−2^ sr^−1^) has been directly correlated with viral titres in mice infected with influenza, Dengue and Sendai reporter viruses^11^,^13^,^14^,^24^,^25^. However, light attenuation by diffusion and scattering in tissue can complicate quantification of *in vivo* bioluminescent imaging and precise localization of the bioluminescent tissue, especially in larger animals. Bioluminescence from firefly luciferase can be imaged as deep as several centimeters within an animal; however, light from shallow tissues can be detected to a greater extent than that from deeper areas where attenuation by overlying tissues can be up to 10-fold^26^,^27^,^28^. NLuc, however, possesses 150-fold greater specific activity (that is, light output) than both *Renilla* and firefly luciferases and may therefore have increased sensitivity and minimized effects due to attenuation^15^. To test this, we correlated bioluminescent flux before and after resection with tissue titres to establish the extent to which overlying tissues attenuate NLuc bioluminescence. Briefly, ferrets (*n*=5) infected with CA/09-PA NLuc virus were imaged, humanely euthanized, breast plates removed and then bioluminescence was immediately measured for a second time (Fig. 3). Infected tissues were recovered and viral titres were determined by TCID_50_. Viral titres from nasal tissue were significantly correlated with flux emitted from this relatively shallow site of replication (Spearman’s *ρ*=0.9910, *P*=0.008) (Fig. 3b). In the lung tissue, increased flux was associated with higher viral titres when data were considered for the whole lung (Fig. 3c,d). Similar results were obtained when analysis was performed on the right or left lobes of the lung, further establishing the linkage between flux and viral titer and allowing more discrete localization of sites of replication. All animals with virus in the lung (as measured by TCID_50_) also displayed flux when resected. Quantitative analyses indicated that a viral load as low as 10^2^ TCID_50_ per gram tissue was sufficient for imaging in intact animals (Fig. 3a,c). Comparison of bioluminescence between whole and resected animals showed that overlying tissue can reduce flux from the lungs up to 4.5-fold (Fig. 3e, ferret 1) and that at least twofold more flux is required to detect bioluminescence in an intact compared with resected animal (Fig. 3f, ferrets 2 and 3). Thus, flux is a highly sensitive measure of viral titre and attenuation of bioluminescence by overlying tissue has a minor impact on the measurement of lung viral loads, although care must be taken with direct comparisons between different replication sites.

### Monitoring real-time influenza virus transmission in ferrets

**Figure 4:**
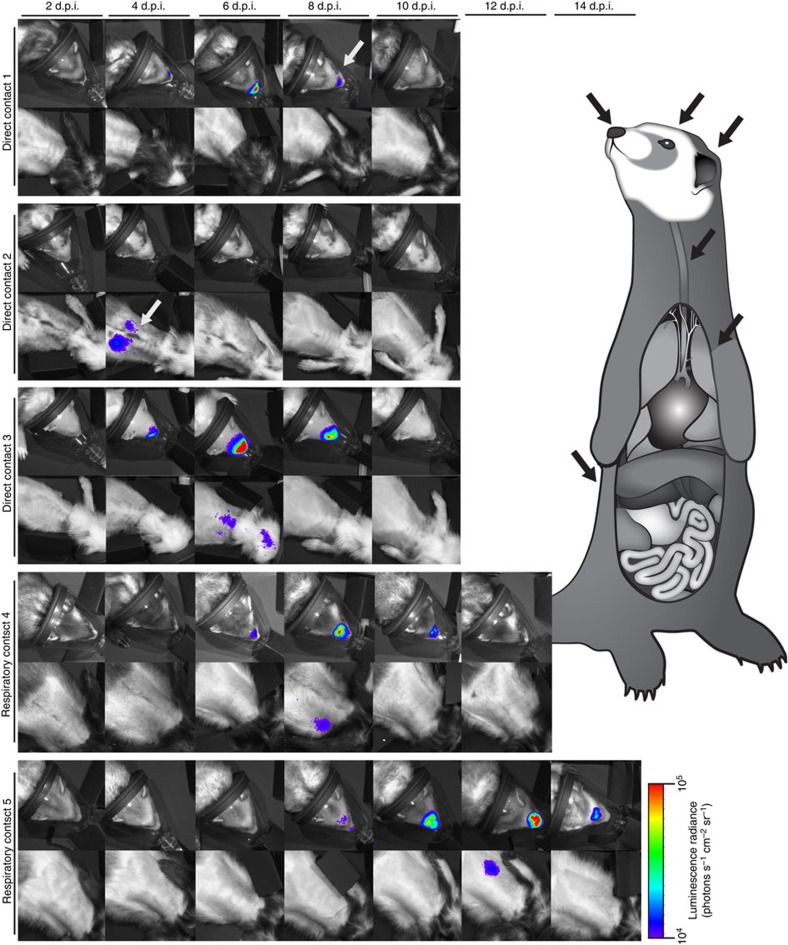
Variable patterns of infection in direct and respiratory contact animals. Naive ferrets were placed in direct contact (*n*=3) or respiratory contact (*n*=2) with inoculated donor animals 1 d.p.i. Upper respiratory and lung bioluminescence was imaged every 48 h.p.i. through 14 d.p.i. Imaging ended when viral clearance was obtained as monitored by both luminescence and viral titre determination. Arrows indicate areas of interest where luminescence was detected.

Having established bioluminescent imaging as a noninvasive and quantitative measure of viral replication, we exploited this system to examine real-time dynamics during influenza virus transmission. Since CA/09 virus is known to transmit by both direct and respiratory contact^21^,^22^,^23^, naive ferrets were placed in the same cages as the inoculated donor ferrets (direct contact, DC) or in cages separated by 4–6 inches from the inoculated group (respiratory contact, RC) at 1 d.p.i. Imaging and viral titres were performed as for the donor ferrets. Transmission occurred in 100% of the DC animals (*n*=3). Bioluminescent flux and viral load peaked at 4–6 d.p.i. with maximum titres reaching ~10^5^ TCID_50_ ml^−1^ in nasal wash (Figs 2b–d and 4). Infections were subsequently detected in all of the RC ferrets (*n*=2), although viral peaks were delayed occurring 8–12 dpi (Figs 2 and 4). Similar to donor animals, luminescence was readily detected directly in the nasal wash of all contact animals supporting rapid analysis of viral shedding (Fig. 2e).

Intriguingly, the spatial resolution of bioluminescent imaging allowed identification of distinct patterns of infection. All of the donor animals displayed at least some degree of replication in both the URT and LRT (Fig. 2a). DC animals displayed highly localized infections in the URT alone (direct contact 1), LRT alone (direct contact 2) and both URT/LRT patterns (direct contact 3, Fig. 4). Nasal wash titres from the DC2 ferret were negative at all time points tested (Fig. 2), although this animal displayed obvious lung bioluminescence. In the absence of bioluminescent imaging, this animal would have been considered uninfected until seroconversion was assessed. In contrast to DC, diversity of tissue tropism was not detected in the RC ferrets where replication occurred in both the URT and LRT (Fig. 4). In spite of the distinct patterns of infection between cohorts, there were no differences in clinical signs including temperature and weight loss (Supplementary Fig. 1) suggesting that the relatively mild CA/09 replication in the lungs may not be associated with more severe clinical disease.

Luminescence was also detected at unexpected sites outside of the respiratory tract, including bioluminescent foci suggesting replication in areas we hypothesize, are the kidney (Fig. 2, donor 3, arrow), the ears (Fig. 2, donor 3, arrow) and the eye or conjunctiva (Fig. 4, direct contact 1, arrow). Although previous studies have shown replication of CA/09 and other influenza viruses in these extra-pulmonary sites^29^,^30^,^31^,^32^, we were unable to euthanize animals to confirm viral replication in these unconventional tissues due to the longitudinal nature of the study. Importantly, results from unbiased whole-body imaging can be used to better understand influenza virus tropism.

### Visualizing immunological protections upon re-challenge

**Figure 5:**
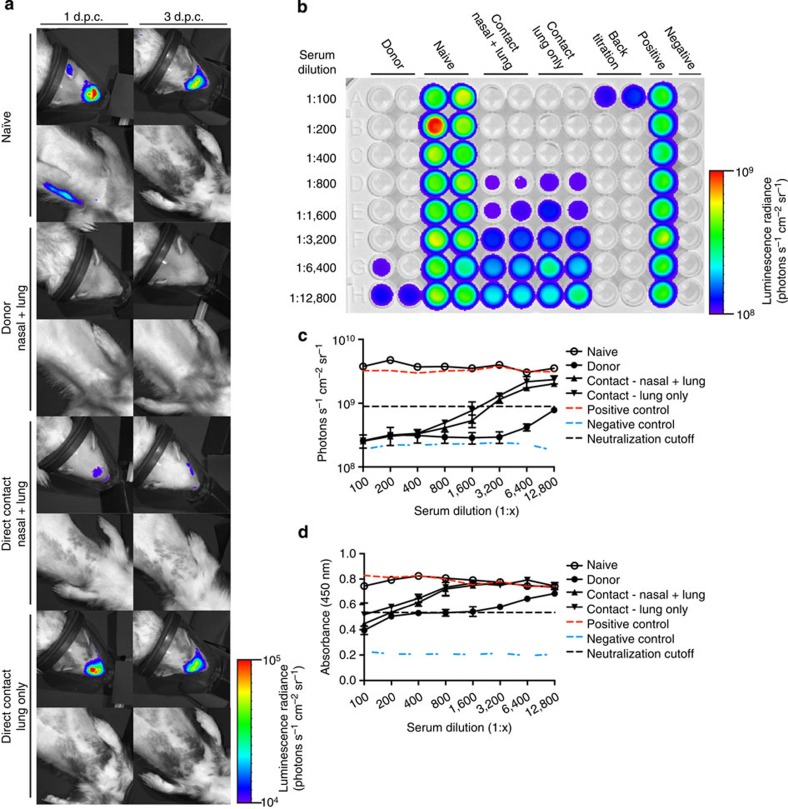
Patterns of infection during primary infection impact the outcome during re-challenge. (**a**) Naive or previously infected animals (*n*=4 per group) exhibiting different patterns of infection were re-challenged with either rgCA/09 or CA/09-PA NLuc virus and images collected at 1 and 3 d.p.c. (**b**–**d**) Microneutralization titres were conducted by luminescent (**b**,**c**) and traditional (**d**) methods on duplicate samples from each animal run in triplicate. Error bars=s.d.

Ferrets are also important models for establishing the efficacy of antiviral therapies and vaccines^7^,^8^. To demonstrate the utility of the NLuc reporter virus for therapeutic studies, naive ferrets or those previously infected with CA/09-PA NLuc were challenged with CA/09-PA NLuc or CA/09 viruses at 28 days post primary infection. Images (Fig. 5a) and nasal washes were collected at 1 and 3 days post challenge (d.p.c.) and nasal turbinates, trachea and lungs collected at 3 d.p.c. Viral titres in naive ferrets inoculated with either the parental rgCA/09 or CA/09-PA NLuc viruses were nearly identical in nasal washes 1 and 3 d.p.c. as well as in the nasal epithelium, trachea and lungs at 3 d.p.c. (Table 2). Although all of the previously infected animals had neutralizing antibodies (Table 2) and were protected from challenge, the ferret that had a primary infection in the lung (Fig. 4, direct contact 2) had measurable virus in the nasal wash, trachea and lungs suggesting that the site of primary infection may impact protection from re-infection (Fig. 5a, direct contact 2). Though this has been shown in infected mice^24^,^33^, further studies are needed to evaluate this in influenza virus-infected ferrets.

Finally, we asked whether the NLuc reporter virus was a more sensitive means to measure neutralizing antibody responses by microneutralization (MN) assay. To test this, sera collected from animals used for the re-challenge studies were assessed for neutralizing antibody titres by classical or luminescent-based MN utilizing the CA/09-PA NLuc virus (Fig. 5b–d). The luminescent-based MN assay showed increased sensitivity with neutralization titres two- to four-times higher using the infectious luminescent assay versus traditional enzyme-linked immunosorbent assay (Fig. 5b–d and Table 2). Interestingly, this increase in sensitivity may represent titres lower than previous methodology was able to detect as well as possible infections by defective particles or semi-infectious virions, which do not productively replicate in the cell.

## Discussion

In summary, our studies demonstrate the utility of the NLuc-based reporter virus to monitor the dynamics of influenza virus transmission as well as viral tropism and therapeutic efficacy studies in the ferret model. Although previous reporter viruses have been described^10^,^11^,^12^,^13^,^14^, our studies are novel by identifying a non-attenuated reporter system that is sufficiently sensitive to be used in a large animal model. These data clearly demonstrate that spatio-temporal dynamics of viral replication in the nasal turbinates and lung correlate with viral titres obtained by traditional methods in a highly specific manner with only minor interference from overlying tissues. Re-imaging of resected animals and determining the viral load in dissected tissues confirmed replication in these sites. Several putative extra-pulmonary sites of luminescence were also detected in proximity to the conjunctiva, the kidney, the trachea and the ear. However, as the animals in the current experiments were followed longitudinally to track the spread and transmission of influenza virus, it was not possible to sample these tissues and confirm that they were indeed sites of active replication. These results open the door to future studies regarding spread of influenza viruses to non-respiratory tissues, where imaging can be used to identify and direct focused analyses of unexpected sites of replication. Previously published influenza reporter viruses do not afford this opportunity due to their attenuation or instability.

In addition to severity and tropism of direct infection, we have also shown that the reporter virus has sufficient stability and sensitivity to monitor transmission via respiratory droplet and direct contact. These results provide an unprecedented tool to understand not only the transmissibility of specific strains but also how influenza viruses transmit between hosts. Previous studies with Sendai virus have shown that respiratory transmission can be dependent on the primary site of infection^25^, therefore, further studies could help to determine the correlation between sites of influenza virus replication (URT versus LRT) and transmissibility. Moreover, this system provides a rapid means to assess viral shed during transmission studies and more quantitatively monitor neutralizing antibody responses. Understanding transmissibility and pathogenicity is paramount not only for newly emerging influenza strains but also for laboratory-generated reassortant strains assessing different aspects of viral fitness and genetic makeup. Therefore, the use of this reporter virus to monitor *in vivo* influenza transmission and pathogenicity in real-time greatly increases the utility of ferret studies by reducing the necessary animal numbers, decreasing the time necessary to make informed experimental decisions on tissue collection and end points, and enhancing sensitivity of assessing current methods of protection and therapeutics. Furthermore, this technology has the potential to be applied to any relevant influenza virus allowing the study of not only seasonal influenza virus but also the highly pathogenic avian influenza virus strains.

The data gained from pathogenicity and transmission studies in ferrets are essential for timely and effective public health measures during an influenza outbreak, for predicting pandemic potential, and for developing effective preventions and interventions. The real-time analysis by our reporter virus system has the potential to accelerate these measurements, and the resulting public health decisions. Since it is impossible to predict which influenza viruses will preferentially emerge and potentially cause the next pandemic, determining the transmissibility of emerging viruses is one of the most important risk-assessment tools when evaluating the potential threat of these viruses. The ability to monitor the dynamics of influenza replication and transmission in real-time in individual animals will be invaluable for risk-assessment studies in addition to evaluating the therapeutic responses against emerging strains.

## Methods

### Reverse genetics and viral propagation

Recently we demonstrated that NanoLuc (NLuc), a 19-kDa engineered luciferase that possesses 150-fold greater specific activity (that is, light output) than both *Renilla* and firefly luciferases^15^, could be stably incorporated into the polymerase subunit (PA) of the WSN virus with no loss of replicative ability *in vitro* and recapitulates known restrictions due to host range and antiviral treatment *in vivo*^16^. Since bioluminescent imaging of viral infections in ferrets has not been reported, we successfully rescued a replication-competent NanoLuc A/California/04/2009 (CA/09-PA NLuc) virus using the eight-plasmid 293T/MDCK co-culture system as described^16^,^19^. Viruses were confirmed by sequence analysis and propagated in the allantoic cavity of 10-day-old specific pathogen-free embryonated chicken eggs at 37 °C. Allantoic fluid was harvested, cleared by centrifugation and stored at −80 °C.

### *In vitro* infections

Infections were performed as described previously^19^. Briefly, MDCK cells were cultured in Eagle’s minimum essential medium (MEM, MediaTech, Manassas, VA) supplemented with 2 mM glutamine and 10% fetal bovine sera (Gemini BioProducts, West Sacramento, CA) and grown at 37 °C under 5% CO_2_. MDCK cells were infected with a multiplicity of infection of 1 for 1 h at 37 °C. Cells were washed three times to remove unbound virus and infected cells were cultured in appropriate media containing 0.075% BSA and 1 μg ml^−1^ TPCK-treated trypsin. Aliquots of culture supernatants were collected at 24, 48 and 72 hours post infection (h.p.i.) and immediately stored at −80 °C for determination of virus titres. No attenuation of luminescence was seen after three passages in eggs and the reporter virus had near-native growth kinetics in MDCK cells as compared with the parental reverse genetics-derived (rg)CA/09 virus with only a slight attenuation at 72 h.p.i. (Fig. 1a).

### TCID_50_ and Nano-Glo TCID_50_ assays

To conduct TCID_50_ assays, MDCK cells were infected with 100 μl of 10-fold serial dilutions of sample and incubated at 37 °C for 72 h. Following incubation, viral titres were determined by hemaglutination assay using 0.5% turkey red blood cells and evaluated by the method of Reed and Muench^19^. Luminescent TCID_50_ assays were modified as follows: MDCK cells were plated on white polystyrene plates (Corning, Corning, NY) and infected with 100 μl of 10-fold serial dilutions of samples as before^19^. Following incubation, 75 μl was removed, plates were frozen at −80 °C and thawed to lyse cells. Prepared Nano-Glo working substrate solution (25 μl) was then added per manufacturer’s instructions and plates were read on a luminometer with gain set 100 to prevent variability between plates, The luminescent assay was optimized to be read within 18 h after titrating virus on MDCK cells as compared with the 72 h required for a traditional TCID_50_ assay (Fig. 1c).

### Hemagglutination inhibition and microneutralization assays

Ferret serum was treated with receptor destroying enzyme (RDE; Seiken) and traditional serological assays were performed according to WHO guidelines^34^. For luminescent microneutralization assay, RDE-treated sera were diluted in microneutralization media (MEM, 2 mM glutamine, 1% bovine serum albumin) at 1:2 dilutions. Diluted sera were then incubated with 100 TCID_50_ virus for 1 h at 37 °C. Following incubation, 3 × 10^4^ MDCK cells were added to each well and plates were incubated at 37 °C overnight. All but 25 μl of overlying media was removed and plates were frozen for >4 h at −80 °C. Luminescence was determined by adding 25 μl Nano-Glo substrate solution (Promega) according to manufacturer’s instructions and the plates were imaged using a Xenogen IVIS200 system with LivingImage software (Xenogen) for 1 s. Neutralization was considered to be any well below the luminescence generated by a well infected with 10 TCID_50_ CA/09-PA NLuc virus.

### Animal experiments

All animal experiments were approved by the St Jude Children’s Research Hospital Animal Care and Use Committee. Donor ferrets (males 8–10 weeks old; Triple F Farms, Sayre, PA) were inoculated intranasally with 10^5^ TCID_50_ units in 1 ml phosphate-buffered saline (PBS). Twenty-four hours later, naive ferrets were placed either in direct contact with the infected group (DC animals) or housed in separate cages 4 to 6 inches from the inoculated group (RC animals)^35^,^36^. A total of five donor ferrets were inoculated, where three were paired with direct contact animals and two with respiratory contact animals. Body weight and temperature were assessed every 24 h and the ferrets were monitored for the following clinical signs: anorexia, sneezing, nasal discharge and lethargy. All CA/09-PA NLuc-infected ferrets utilized for the study were imaged and are shown in the figures.

Nasal washes were collected at every 2 d.p.i. for viral titration and sera were collected at 14 d.p.i. for HI analysis, as described^35^,^36^. To collect nasal washes, the ferrets were anaesthetised intramuscularly with 0.25 to 0.3 ml of ketamine solution (30 mg kg^−1^ of body weight ketamine) and sneezing was induced by the drop-wise addition of 1 ml of sterile PBS containing antibiotics (100 U ml^−1^ penicillin, 100 μg ml^−1^ streptomycin) to each nostril. Nasal washes were collected in a sterile specimen cup and viral titres were determined by TCID_50_ analysis.

For bioluminescent imaging, animals were shaved in the area over the lung to minimize background. At each time point, animals were anaesthetised with isoflurane, then given an intravenous injection of 200 μl (~0.3 μl g^−1^ weight) Nano-Glo substrate (Promega) diluted in 900 μl sterile PBS via cephalic route. In some cases, saphenous or jugular route had to be utilized for injection; however, these secondary injection sites provided efficient means of delivering subrate for bioluminescent imaging. Anaesthesia was maintained throughout by nasal delivery of isoflurane. Animals were immediately imaged for 4 min using a Xenogen IVIS200 system with LivingImage software (Xenogen). Due to machine size and animal orientation, two images were necessary to obtain data for both the upper respiratory tract and lungs. Fresh substrate injections were required before each image and ~8–10 min elapsed between injections. Ferrets inoculated with rgCA/09 showed no detectable luminescence (Fig. 1b). No differences were observed in luminescence due to the route of injection.

The limit of detection of the CA/09-PA NLuc virus in ferret tissue and localization of bioluminescent foci to discrete tissues was performed by infecting five additional ferrets. Animals were imaged 3 d.p.i., resected and immediately re-imaged. Nasal and lung tissues were collected and tissue titres were determined. For re-challenge experiments, naive or previously infected ferrets were inoculated with 10^5^ TCID_50_ CA/09-PA NLuc and imaged at 1 and 3 d.p.c. At 3 d.p.c., animals were euthanized and nasal turbinates, trachea and lungs were harvested for titres. Serum was also collected for microneutralization assay.

## Author contributions

E.A.K., V.A.M., A.M. and S.S.-C. designed and planned the project. E.A.K., V.A.M., C.S. and B.L. performed the experiments. E.A.K., V.A.M., A.M. and S.S.-C. analysed the data, discussed the results and wrote the paper. All the authors commented on the manuscript.

## Additional information

**How to cite this article:** Karlsson, E. A. *et al.* Visualizing real-time influenza virus infection, transmission and protection in ferrets. *Nat. Commun.* 6:6378 doi: 10.1038/ncomms7378 (2015).

## Supplementary Material

Supplementary Information Supplementary Figure 1

## Figures and Tables

**Table 1:** Correlation between titres obtained by different methodologies and comparing rgCA/09-PA NLuc with rgCA/09 parental virus.

Correlation*								
**Ferret**	**Wash**† **versus luminescent titre**‡		**Wash versus traditional titre**§		**Luminescent titre versus traditional titre**		**Traditional titre versus parental titre**||	
	***r***	***P***	***r***	***P***	***r***	***P***	***r***	***P***
Donor 1	0.6772	0.0451	0.7293	0.0258	0.867	0.0025	0.8125	0.0142
Donor 2	0.8086	0.0083	0.8321	0.0054	0.9202	0.0004	0.8109	0.0146
Donor 3	0.7892	0.0114	0.8521	0.0035	0.9296	0.0003	0.8116	0.0144
Donor 4	0.7384	0.0231	0.8252	0.0062	0.9246	0.0004	0.8281	0.0111
Donor 5	0.7405	0.0225	0.7564	0.0183	0.9265	0.0003	0.8617	0.0059
Direct contact 1	0.7401	0.0226	0.7701	0.0152	0.9365	0.0002	0.9778	>0.0001
Direct contact 2	1	>0.0001	1	>0.0001	1	>0.0001	1	>0.0001
Direct contact 3	0.7291	0.0258	0.7347	0.0241	0.9304	0.0003	0.9533	0.0002
Aerosol 1	0.9128	0.0006	0.9742	<0.0001	0.9809	<0.0001	0.8953	0.0027
Aerosol 2	0.71	0.0321	0.754	0.0189	0.9946	<0.0001	0.8687	0.0051

^*^Titres for each individual animal were compared using Pearson’s correlation (*α*=0.05).

^†^Luminescence read directly from nasal wash without titrating on MDCK cells.

^‡^Modified TCID_50_ assay on MDCK cells read by luminescence after 16 h.p.i.

^§^Traditional TCID_50_ assay on MDCK cells read by hemagglutination after 72 h.p.i.

^||^Comparison of traditional TCID_50_ assays between ferrets infected with CA/09-PA NLuc and rgCA/09 viruses.

**Table 2:** Serology, nasal wash and tissue titres in virus re-challenged ferrets.

**#**	**Group**	**Site of infection**	**HAI titer**	**MN titer**	**Re-challenge virus**	Titres post challenge*			
						**Nasal wash**	**Nasal**†	**Trachea**†	**Lung**†
1	Naive	NA	0	0‡	rgCA/09	10^5.25^ §	10^5.25^	10^3.5^	10^4^
				0||		10^4^ ¶			
2	Naive	NA	0	0	CA/09-PA NLuc	10^5.5^	10^5.5^	10^3.5^	10^3.75^
				0		10^3.5^			
3	Donor 1	Nasal and lung	1,280	1:1,600	CA/09-PA NLuc	0	0	0	0
				1:12,800		0			
4	Direct contact 3	Nasal and lung	320	1:200	CA/09-PA NLuc	0	0	0	0
				1:2,400		0			
5	Direct contact 2	Lung only	320	1:100	CA/09-PA NLuc	10^4.5^	10^3.75^	10^1^	10^2.5^
				1:1,600		10^2.5^			

NA, not applicable.

^*^TCID_50_ per ml.

^†^Three days post challenge only.

^‡^Enzyme-linked immunosorbent assay end point.

^§^One day post challenge.

^||^Luminescent end point.

^¶^Three days post challenge.
