# Supplementary material for: Visualizing real-time influenza virus infection, transmission and protection in ferrets
Source: Nat Commun. 2015 Mar 6;6:6378. doi: 10.1038/ncomms7378 (PMC4366512; doi:10.1038/ncomms7378)
Supplement: Supplementary Information — Supplementary Figure 1 [file ncomms7378-s1.pdf]

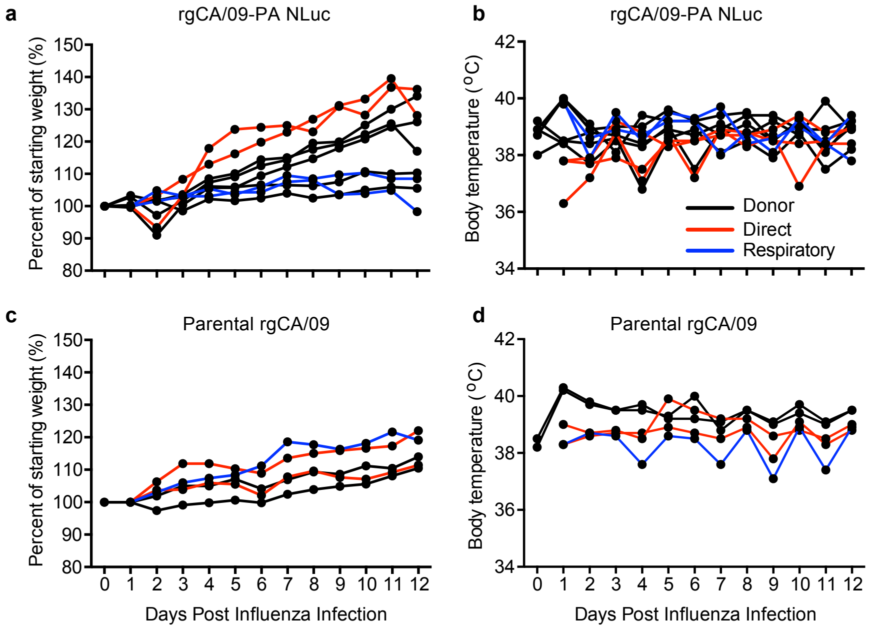

Supplementary Figure 1. Clinical comparisons of animals infected with CA/09-PA NLuc or parental rgCA/09 influenza virus. Eight-week-old male ferrets were lightly anesthetized and intranasally inoculated with 10^5 $TCID_{50}$ CA/09-PA NLuc or rgCA/09 virus. CA/09-PA NLuc infected animals did not show significant (a) weight loss or (b) body temperature increase. Data represents 9 animals (5 donor, 2 direct contact, 2 respiratory contact). Each line represents an individual animal. Similarly, parental rgCA/09 infected animals did not show significant (c) weight loss or (d) body temperature increase. Data represents 5 animals (2 donor, 2 direct contact, 1 respiratory contact). Each line represents an indivdual animal.
